# Bipolar-assisted aneurysm remodeling in microsurgical clipping: safety profile, technical applications, and clinical outcomes

**DOI:** 10.1007/s00701-026-06838-y

**Published:** 2026-04-13

**Authors:** Mehmet Sabri Gurbuz, Ece Uysal, Hidayet Safak Cine, Yunus Emre Ozbilgi, Mohammed Aladdam, Gianluca Lorenzo Fabozzi, Abuzer Gungor, Sabino Luzzi

**Affiliations:** 1 Istanbul Medeniyet University, Istanbul, Türkiye; 2 Acibadem Altunizade Hospital, Istanbul, Türkiye; 3 University of Sassari, Sassari, Sardinia, Italy; 4 Azienda Ospedaliero Universitaria Sassari, Sassari, Sardinia, Italy; 5 Istinye University, Istanbul, Türkiye

**Keywords:** Aneurysm remodeling, Bipolar coagulation, Dome and neck modification, Intracranial aneurysm, Microsurgical clipping, Aneurysmorrhaphy

## Abstract

**Purpose:**

Bipolar coagulation was historically described as a method for dome or neck remodeling, but concerns about rupture kept it underused. Yet carefully applied, low-power bipolar bursts can safely shrink or stiffen aneurysm walls, improving clip access in select challenging cases. This study aims to systematically evaluate how bipolar coagulation is used during intracranial aneurysm clipping, including its indications, intraoperative roles, and impact on surgical exposure and postoperative outcomes.

**Methods:**

We retrospectively analyzed 50 consecutive patients who underwent microsurgical clipping of intracranial saccular aneurysms in which bipolar coagulation was deliberately employed. High-resolution surgical videos were reviewed to document the timing (pre-clip vs post-clip), target (dome vs neck), and purpose (remodeling, shrinkage, dissection assistance, or repair). Aneurysm morphology, rupture status, clip strategy, and perioperative variables were obtained from operative and radiological records. Patients were monitored clinically and radiographically at standardized intervals up to 24 months.

**Results:**

Bipolar techniques were applied for neck remodeling in 31 patients (62%), dome coagulation in 30 patients (60%), post-clip coagulation in 29 patients (58%). Sole bipolar coagulation combined with cotton wrapping was used in 1 patient (2%). No statistically significant differences were observed among groups in preoperative WFNS scores (*p* = 0.20), discharge WFNS scores (mean 1.38 ± 1.07; *p* = 0.71), number of clips used (*p* = 0.56), mortality (*p* = 0.93), or indication distribution (*p* = 5.45). Early postoperative imaging demonstrated complete aneurysm obliteration in 48 of 50 patients (96%), while residual aneurysm was detected in 2 patients (4%). Overall mortality during follow-up was 6% (3/50), with no deaths attributable to surgical technique; all deaths were related to vasospasm.

**Conclusion:**

Selective, low-power bipolar coagulation was used as an adjunct during aneurysm clipping without bipolar-related intraoperative rupture in this series. Its controlled use can enhance visualization, optimize clip positioning, and expand treatment options in anatomically challenging aneurysms.

**Supplementary Information:**

The online version contains supplementary material available at 10.1007/s00701-026-06838-y.

## Introduction

Intracranial aneurysm surgery has greatly advanced with the advent of microsurgical techniques. A key innovation was the integration of the microscope and microinstruments into neurosurgery followed by the invention of bipolar coagulation forceps, first pioneered in the 1950 s by Leonard Malis and soon adopted by M. Gazi Yasargil in the 1960s under the operating microscope. Yasargil, in the first page of his book Microneurosurgery volume IVB, denoted that with the microscope alone- without bipolar coagulation- microneurosurgery would not attain its current standards. This tool enabled safe tissue manipulation and refined hemostasis without coagulating the normal tissue, transforming previously inoperable cerebrovascular lesions into treatable ones. Yasargil described using bipolar electrocautery in early aneurysm clipping procedures as an adjunct to secure clipping in 1969 and published in his book excellent illustrations revealing how he performed bipolar coagulation to facilitate a safer aneurysm clipping [19, 21]. By the 1980 s, Japanese surgeons introduced an “aneurysm dome coagulation” technique to shrink the sac and facilitate clip application. Sano et al. reported this method and its histopathological effects in 1984, and later Kato et al. detailed technical pitfalls and refinements of intraoperative cautery in acute aneurysm surgery [9, 17]. However, due to concerns about intraoperative rupture, such direct coagulation of aneurysm domes remained a cautiously used and under-documented technique for years [8, 15].

Despite limited early adoption, various groups demonstrated the value of judicious bipolar coagulation in select cases. Bipolar cautery has even been applied to difficult-to-clip microaneurysms as an alternative to clipping. Nussbaum et al., for example, reported obliterating tiny intracranial microaneurysms with bipolar electrocoagulation combined with parent vessel reinforcement, avoiding more invasive bypass or trapping procedures [14]. These experiences suggested that controlled bipolar coagulation can effectively shrink or thrombose aneurysm sacs that are not amenable to standard clipping.

Microsurgical masters further refined these techniques. Choque-Velasquez J et al., advocated a “short-burst” bipolar coagulation method as part of aneurysm surgery. They also employed strategic dome cauterization in certain cases – sometimes nicknamed “killing the aneurysm” – wherein after clipping the neck, the aneurysm dome is coagulated and excised to ensure complete obliteration (particularly helpful in complex or giant aneurysms) [4]. Kulwin et al., described a modern dome-shrinkage technique (“aneurysmorrhaphy”) for broad-based middle cerebral artery aneurysms [10]. In this approach, the aneurysm dome is partially coagulated (with low current under saline irrigation) to reduce its volume and improve the surgeon’s view of the neck, thereby facilitating clip placement [6]. Even very small (< 3 mm) aneurysms that pose clipping challenges have been successfully managed in experienced centers using advanced methods [2, 7].

Building on this background, the present study aims to evaluate the intraoperative use of bipolar coagulation in microsurgical aneurysm clipping. The study also provides insight into the stepwise and controlled introduction of bipolar coagulation techniques within a standardized microsurgical learning framework. We will analyze how frequently and for what specific purposes bipolar cautery is employed during aneurysm surgery (e.g. neck remodeling, dome reduction, bleeding control), and assess its impact on surgical efficacy and patient outcomes. By elucidating the roles and results of this technique, we hope to inform its optimal application and claim it’s safety in the management of intracranial aneurysms.

## Materials and methods

### Study design and patient population

This study is a single-center retrospective observational analysis of patients who underwent microsurgical intracranial aneurysm clipping with the adjunct use of bipolar coagulation. We reviewed 50 consecutive ruptured and unruptured aneurysm patients treated by a single experienced cerebrovascular neurosurgeon between February 2023 and July 2025 at our institution. The study was approved by the Institutional Review Board of Istanbul Medipol University (Approval No: 1180, Date: 28.11.2024), and all procedures adhered to the Declaration of Helsinki. All intracranial saccular aneurysm cases in which bipolar coagulation was used at any stage of surgery (pre-clip, post-clip, dome, neck, or repair-oriented coagulation) were included, encompassing both ruptured (aneurysmal subarachnoid hemorrhage) and unruptured aneurysms.

### Inclusion and exclusion criteria

Eligible cases included adult patients (> 18 years) who underwent microsurgical clipping of an intracranial saccular aneurysm in which bipolar coagulation was used at any intraoperative stage. Only cases in which bipolar coagulation was deliberately employed were included. It is worth noting that the study did not include a control group of aneurysms clipped without bipolar coagulation. Inclusion required the availability of complete operative video recordings and postoperative imaging studies to ensure accurate assessment of the timing, purpose, and effects of bipolar application. Patients treated exclusively with endovascular techniques, as well as those who underwent microsurgical clipping without the use of bipolar coagulation, were excluded. Additional exclusion criteria included fusiform, dissecting, traumatic, or infectious aneurysms, and cases with incomplete operative documentation or insufficient clinical follow-up. For patients harboring multiple aneurysms, eligibility was maintained if bipolar coagulation had been applied to at least one aneurysm during the same surgical procedure.

### Data collection and variable definitions

Patient demographics (age, gender, comorbidities, smoking history) and clinical presentation (Fisher scale on admission computed tomography (CT), and World Federation of Neurosurgical Societies (WFNS) grade at admission and discharge were recorded. Aneurysm characteristics included location, laterality, maximum dome diameter (mm), and morphological classification (regular vs. irregular/bulbous/lobulated). Rupture status at presentation was documented.

All intraoperative and perioperative variables—including surgical approach, number of permanent clips applied, temporary clip usage, aneurysm rupture or oozing, dome puncture, dome excision, and postoperative residual sac—were obtained from operative reports, imaging studies, and the hospital's digital medical record system.

### Operative video review and aneurysm morphology definitions

All aneurysm cases underwent a meticulous review of high-resolution operative videos to ensure accurate characterization of bipolar coagulation use. Each recording was examined to identify the exact timing of coagulation (pre-clip vs. post-clip), the anatomical target (neck or dome), and the underlying purpose—whether for neck or dome remodeling, sac shrinkage, dissection assistance, or repair of an arterial wall defect. The duration and frequency of each bipolar application were also documented, allowing precise categorization of coagulation type for every case. Additionally, aneurysm morphology was standardized using specific definitions: a “baby aneurysm”, focal outpouching measuring ≤ 2 mm without a discernible neck and considered high-risk for conventional clipping, whereas a bulbous or lobulated aneurysm referred to a sac with an irregular contour or multilobulated dome that obscured the aneurysm neck and restricted surgical exposure.

### Bipolar coagulation categories

**Fig. 1:**
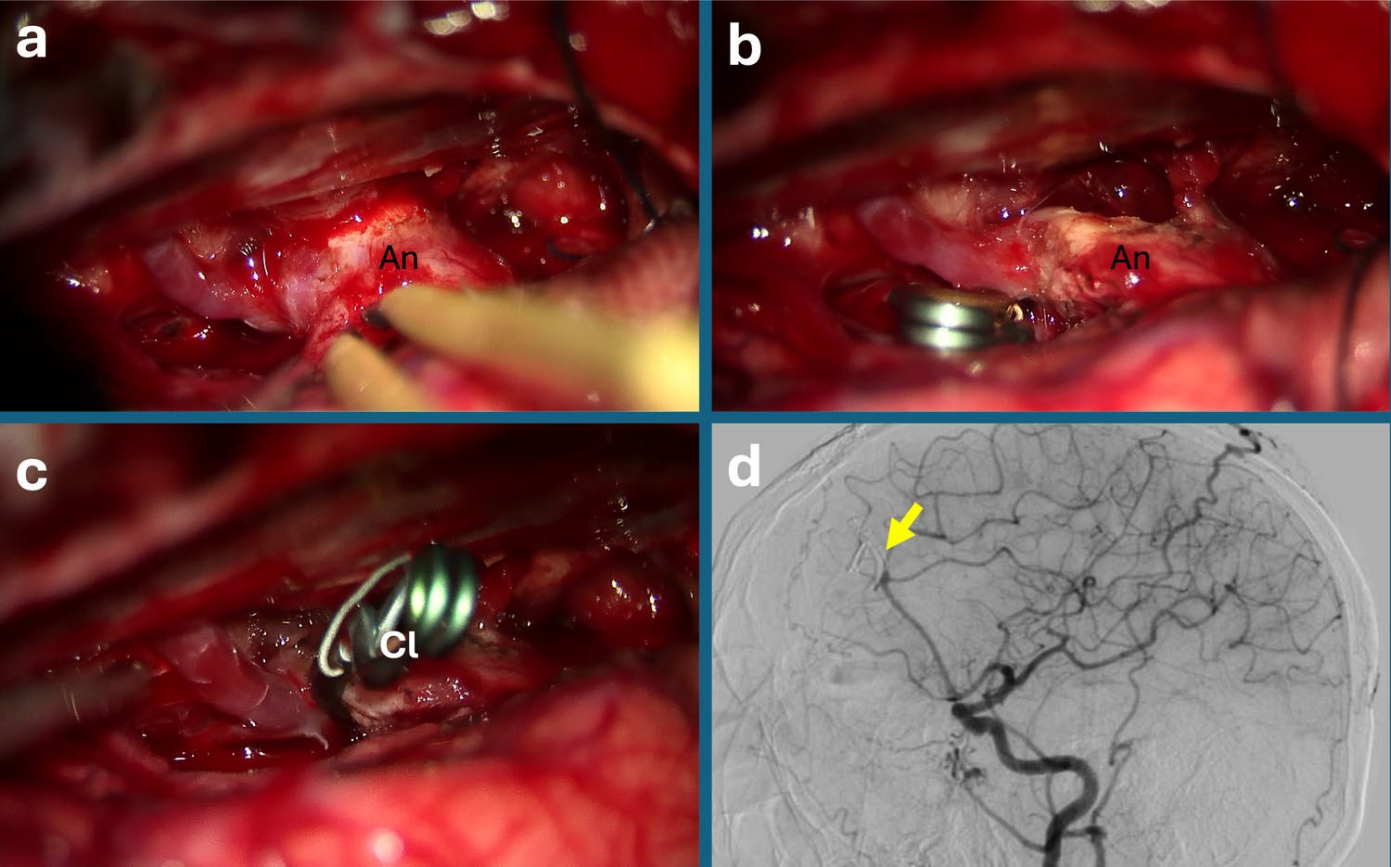
Pericallosal artery aneurysm in a patient with osteogenesis imperfecta. **a** Intraoperative view demonstrating a broad-necked pericallosal artery aneurysm. Given the morphology, the goal was to remodel and narrow the neck—rather than the dome—using bipolar coagulation to achieve a more favorable geometry for clip application. **b** Following targeted neck coagulation, the aneurysm neck becomes reshaped and sufficiently compact to allow safe and effective clip placement. **c** Final intraoperative appearance after permanent clip application, showing complete exclusion of the aneurysm. **d** Postoperative digital subtraction angiography (DSA) confirms patency of both A2 segments with no residual aneurysm filling. Abbreviations: An: Aneurysm; Cl: Permanent Clip
B Pre-clip dome shrinking: Bipolar cautery was used on the aneurysm dome before clip placement to reduce the sac volume and facilitate dissection around the aneurysm. This technique was especially helpful for aneurysms with bulbous domes that obscured the neck or for extremely small “baby” aneurysms. By partially collapsing a turgid dome, the surrounding arterial anatomy could be better visualized and the clip could subsequently be applied more easily to the neck (Figs. 2 and 3, Video 2, 3).

Bipolar Coagulation: We categorized the role of intraoperative bipolar cautery for aneurysm management into four distinct applications, defined as follows:

A Pre-clip neck remodeling: Bipolar coagulation was applied in short, focused bursts directly to the aneurysm neck prior to clip application. This maneuver aimed to slightly shrink or stiffen the neck tissue, creating enough space or a small groove for the clip blades to seat securely. It was used selectively in cases with broad-necked aneurysms where initial clipping angles were unfavorable, to gently modify the neck configuration without fully obliterating the sac (Fig. 1, Video 1).

**Fig. 2:**
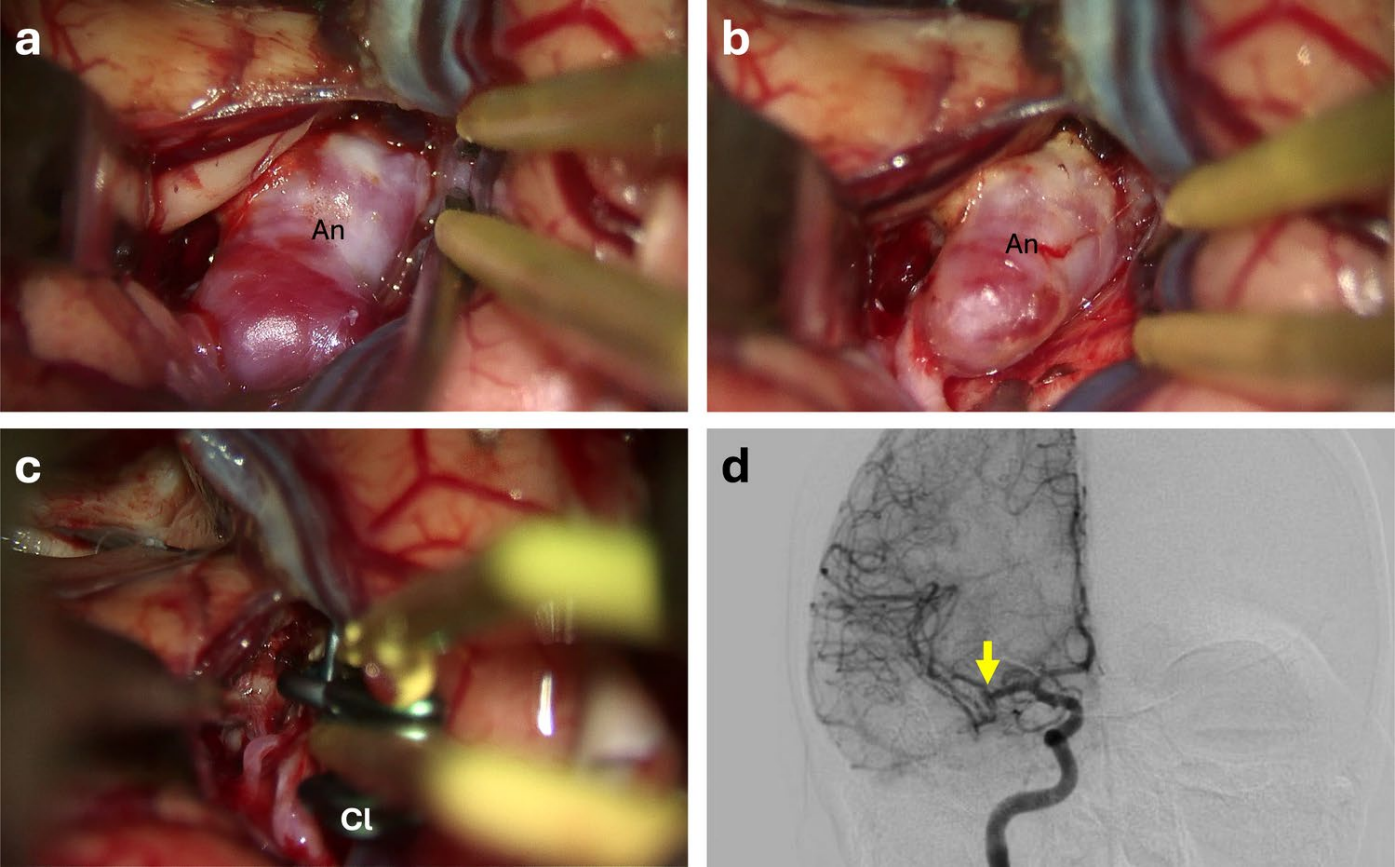
Images of an unruptured MCA aneurysm before and after dome coagulation. **a** Due to the large size of the aneurysm, the surrounding MCA branches and trunk cannot be clearly visualized, and mobilization or safe circumferential dissection is not feasible. **b** Following bipolar dome coagulation, the aneurysm sac becomes significantly smaller and more compact, allowing easier mobilization and dissection from the surrounding arachnoid planes. This maneuver enables clear visualization of the MCA trunk and all adjacent branches. **c** Complete and durable occlusion of the aneurysm is achieved using a straight permanent clip in combination with a fenestrated clip, providing full anatomical reconstruction of the parent vessel. **d** Postoperative digital subtraction angiography (DSA) confirms total aneurysm occlusion without any residual filling. Abbreviations: An: Aneurysm; Cl: Permanent Clip

**Fig. 3:**
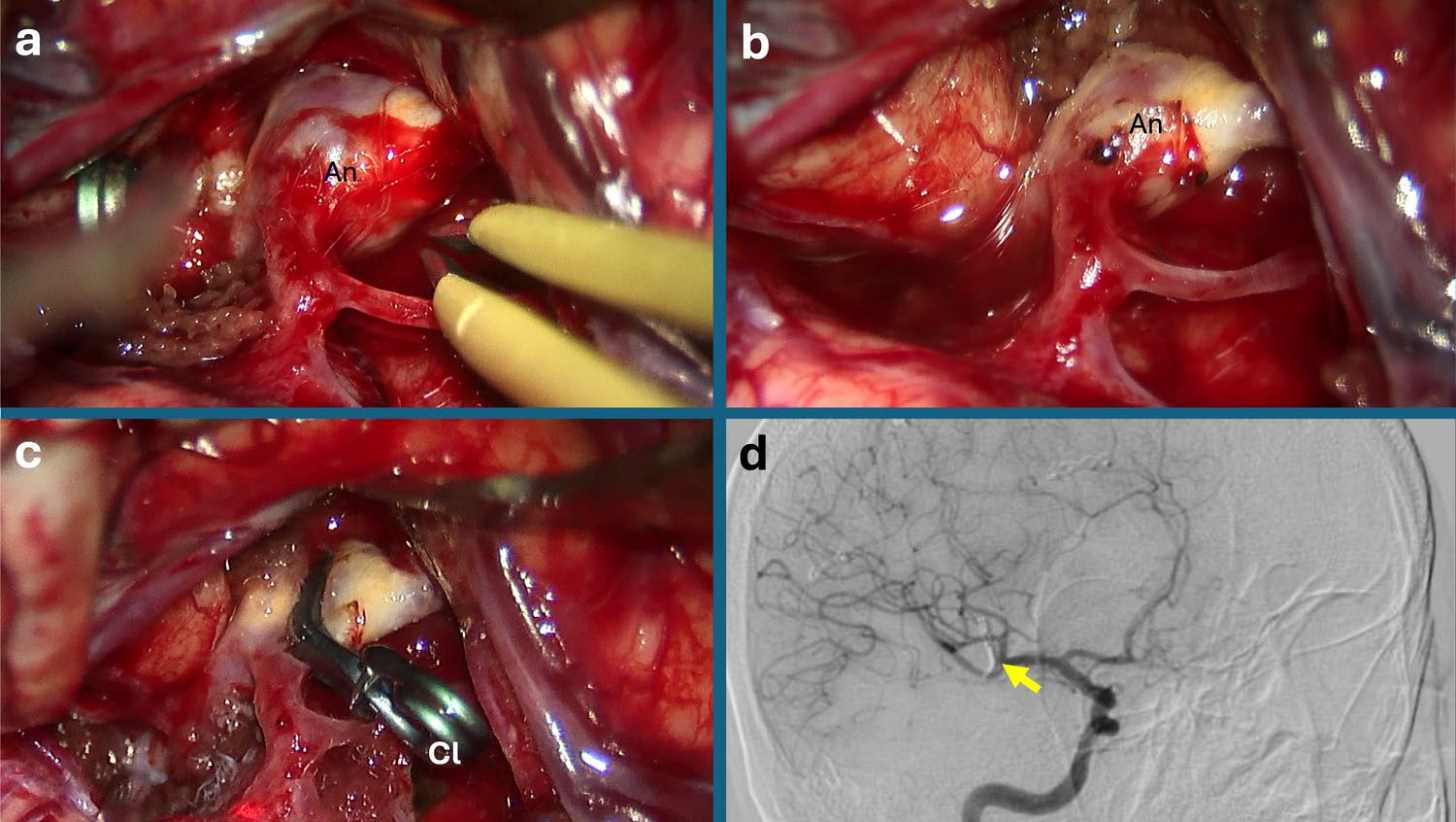
Images of an unruptured MCA aneurysm in a male patient undergoing dome coagulation. **a.** Preoperative microscopic view demonstrating an unruptured middle cerebral artery (MCA) aneurysm. **b.** After bipolar dome coagulation, the aneurysm sac is visibly reduced and prepared for clip application, facilitating a safer and more controlled clipping. **c.** Following clip placement, a calcified-appearing portion of the aneurysm adjacent to the clip is observed; micro-Doppler assessment confirms the absence of residual flow. **d.** Postoperative digital subtraction angiography (DSA) shows complete aneurysm occlusion with no residual filling. Abbreviations: An: Aneurysm; Cl: Permanent Clip
C Post-clip dome remodeling: After definitive clip deployment, bipolar coagulation was applied to the residual aneurysm dome or fundus (between or just beyond the clip blades) to collapse any remaining sac tissue. This well-described maneuver of shrinking the dome after tentative clipping was used to confirm that the aneurysm was completely obliterated and to ensure the clip blades were optimally positioned. Post-clip cauterization helped eliminate redundant dome portions and provided immediate feedback on clip efficacy by checking for any persistent filling or expansion of the sac. All clipped domes, whenever the clipped dome is sizeable to puncture, were punctured and cut (if possible) to ensure the exclusion of the aneurysm from the circulation. If the aneurysm was still filling, the punctured hole was coagulated and secured followed by clip repositioning or additional clip placement until the perfect clipping was attained. Therefore, coagulation of the punctured hole was listed under this category (Fig. 4, Video 4).

**Fig. 4:**
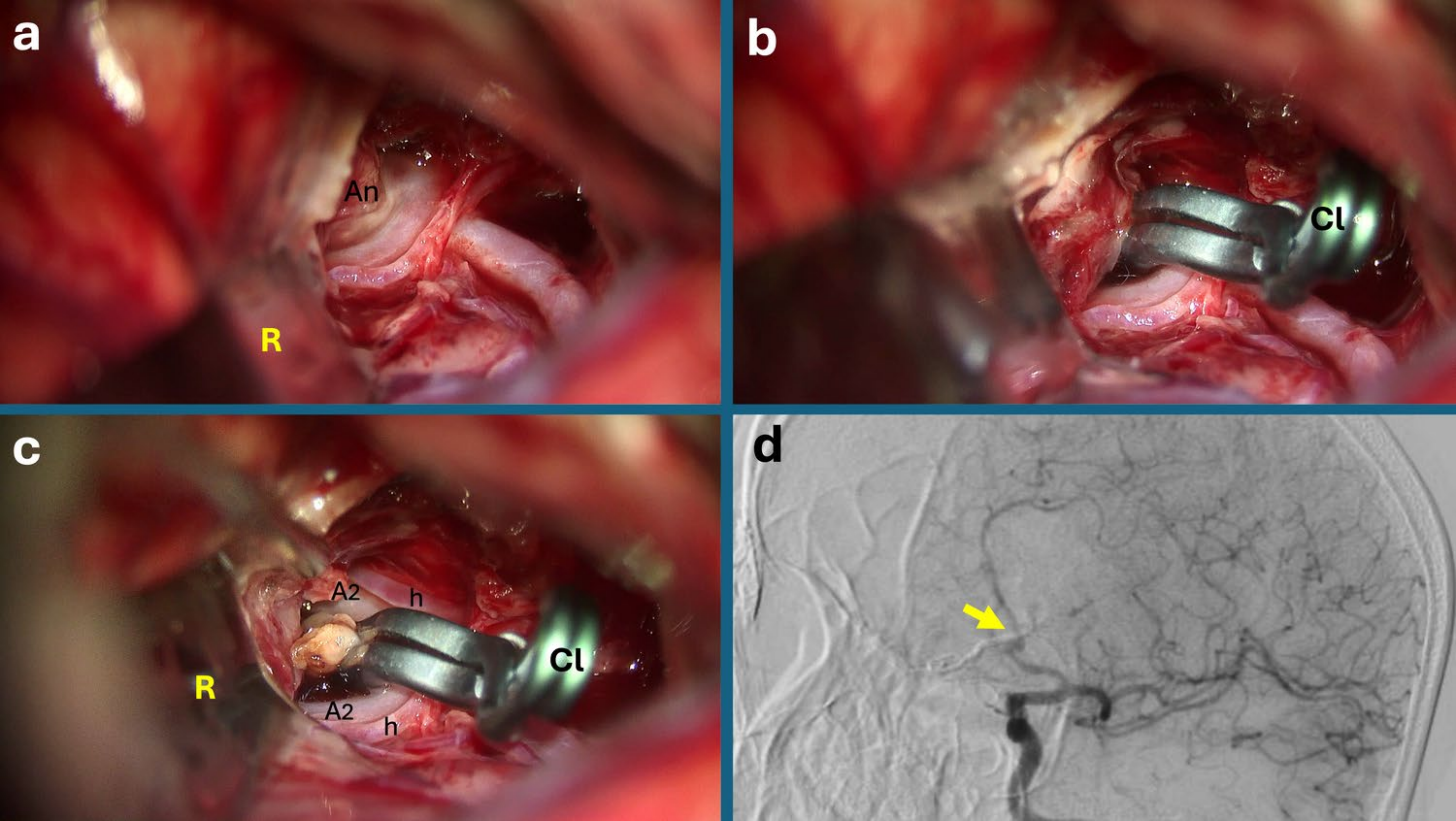
Anterior communicating artery (AComA) aneurysm demonstrating post-clip bipolar coagulation. **a** After retractor placement, the aneurysm is visualized at the AComA complex. **b** Permanent clip application is performed across the aneurysm neck. **c** Following clip placement, bipolar coagulation is used to shrink and remodel the residual aneurysm dome; both Recurrent Artery of Heubner branches and both A2 segments remain clearly patent throughout the maneuver. **d** Postoperative digital subtraction angiography (DSA) confirms preserved bilateral A2 flow and complete aneurysm occlusion with no residual filling. Abbreviations: An: Aneurysm; Cl: Permanent Clip; R: Retractor; h: Heubner
D Only-coagulation/cotton-wrapping: In select cases of extremely small aneurysms that were not amenable to safe clipping, the primary treatment consisted of bipolar coagulation of the aneurysm itself, followed by reinforcement of the aneurysm wall with cotton wrapping. In this scenario, no aneurysm clip was placed; instead, bipolar coagulation achieved aneurysm wall shrinkage and thrombosis, and a cotton pad was laid over the cauterized aneurysm dome for additional wall support. This technique served as a salvage option for very small aneurysms and has been reported as an alternative strategy for delicate micro-aneurysms (Figs. 5 and 6, Video 5). Bipolar coagulation repair of any small arterial wall injury was also mentioned under this category although it is not a maneuver directly related to aneurysm clipping (Video 6).

**Fig. 5:**
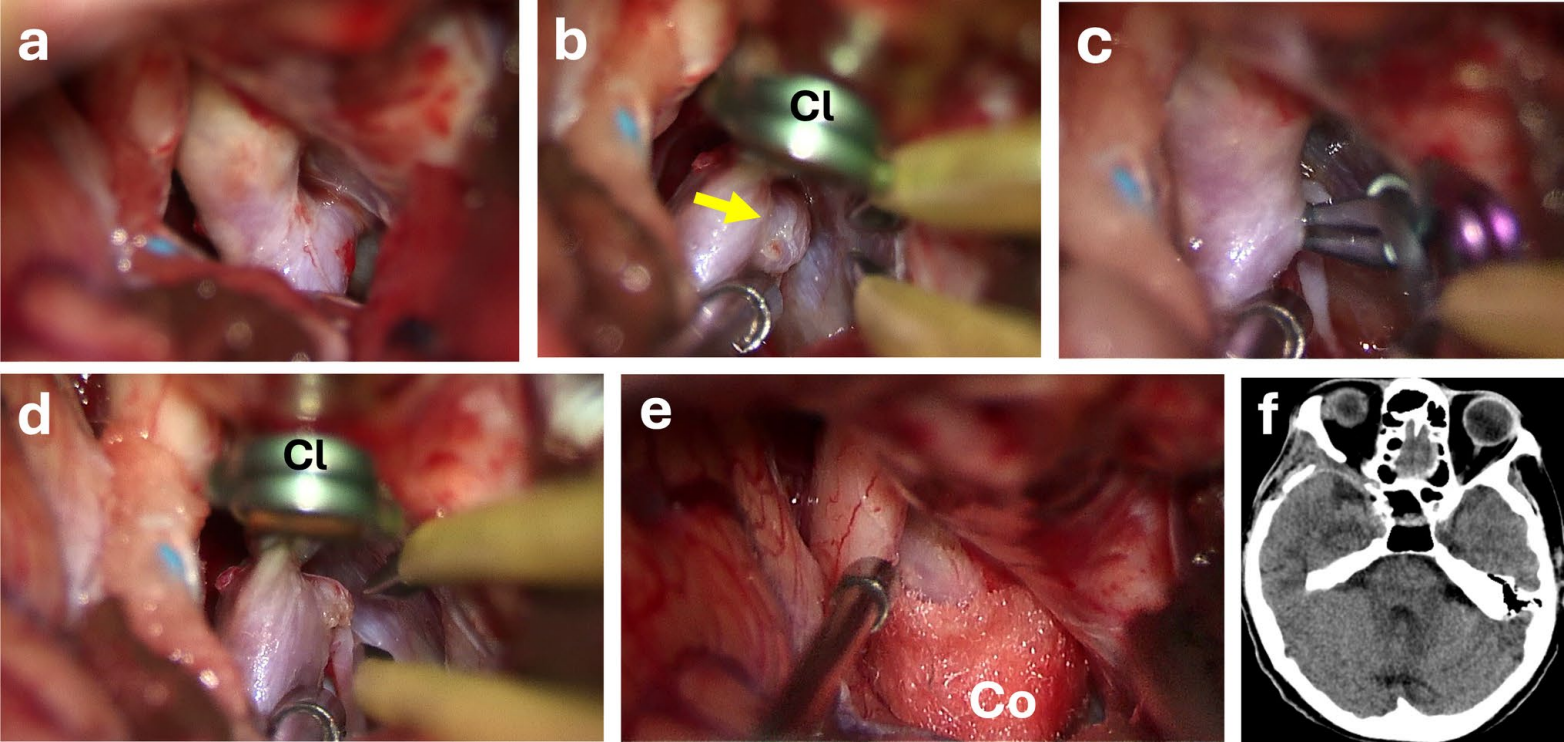
Unruptured aneurysmal dilation at the PComA origin without a true definable neck. **a** Initial intraoperative view shows a broad, ill-defined aneurysmal expansion at the posterior communicating artery (PComA) origin, with no clear neck suitable for direct clip placement. **b** After applying a temporary clip to the parent vessel, bipolar coagulation is initiated to sculpt and create a more distinct neck configuration. **c** Although a neck-like structure appears after coagulation, clip application is not feasible without compromising the PComA; indeed, attempted clipping results in loss of filling in the PComA, making clipping unsafe. **d** The aneurysmal dilation is then completely obliterated using coagulation alone, achieving definitive exclusion without applying a permanent clip. **e** Cotton was applied to reinforce the adjacent arterial wall, providing additional structural support at the site of the treated aneurysmal segment. **f** Postoperative DSA could not be obtained because the patient lacked available relatives for consent and was unwilling to proceed; postoperative CT imaging confirms that no permanent clip was placed. Abbreviations: An: Aneurysm; Cl: Permanent Clip.; Co: Cotton

**Fig. 6:**
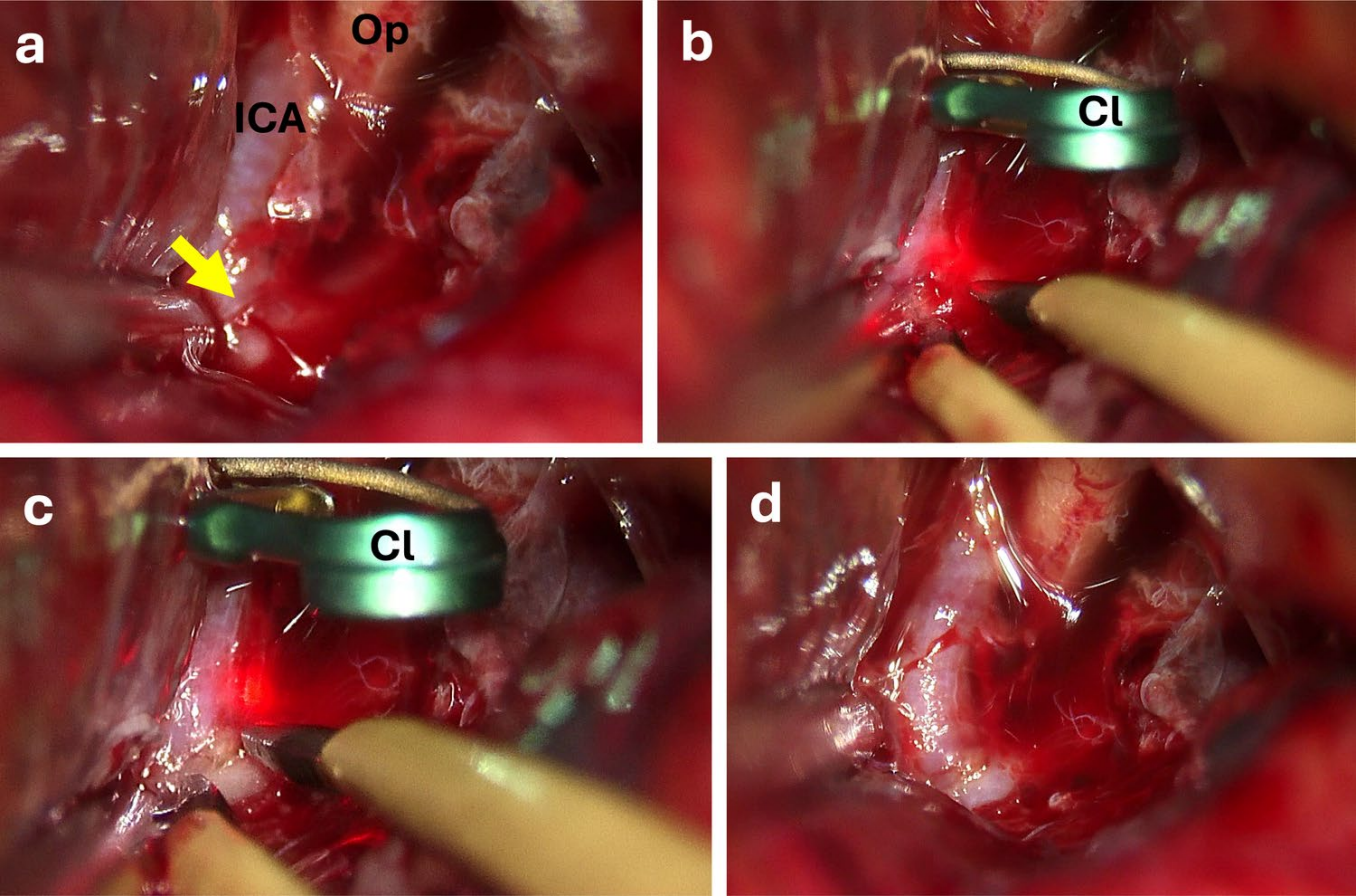
Example of bleeding from the wall of the internal carotid artery (ICA). **a** At the beginning of the sylvian dissection, active bleeding is observed originating from the ICA wall near the bifurcation. **b** After placing a temporary clip, intraluminal pressure is reduced, allowing for controlled visualization of the bleeding point. **c** Bipolar coagulation is applied directly to the arterial wall to achieve hemostasis. **d** Following removal of the temporary clip, the arterial wall appears intact, and no further bleeding is observed. Abbreviations: An: Aneurysm; Cl: Temporary Clip; ICA: Internal cerebral artery; Op: Optic Nerve

Each aneurysm case was assigned one of the above categories based on the intraoperative use of bipolar coagulation. Categories (a) through (c) could be used in combination for a given case if needed (for instance, both pre- and post-clip coagulation in the same surgery), but for descriptive purposes the primary use was recorded.

### Surgical technique

All procedures were performed by a single experienced cerebrovascular neurosurgeon, minimizing inter-surgeon variability. Standard microsurgical approaches were used depending on aneurysm location. All bipolar coagulation applications were performed under temporary clipping of the parent vessel. A temporary clip was placed on the parent artery whenever bipolar cautery was applied, and each temporary occlusion was limited to a maximum of 3 min to minimize the risk of ischemia. Throughout every bipolar application, continuous irrigation with saline was used to cool the tips and target tissue, preventing thermal injury to surrounding structures and avoiding the forceps tips sticking to the aneurysm wall. Importantly, the bipolar forceps used had blunt shoulder tips rather than needle-point tips, and the broad side of the tip (the shaft near the tip) was applied to the aneurysm surface instead of puncturing it. This method concentrated the coagulation effect superficially on the aneurysm wall and minimized the risk of inadvertently perforating the sac.

We refined the bipolar electrocautery power settings during the early part of the study. Initial trial settings of 3, 5, and 7 on the bipolar coagulation unit (Aesculap GN060; Aesculap, Tuttlingen, Germany) were tested in a few cases. Based on these trials, a setting of 7 (approximately corresponding to 10–20% of the device’s maximum power output) was determined to be optimal for achieving effective aneurysm shrinkage without excessive thermal spread. Thus, for the majority of cases, the bipolar generator was set to level 7, delivering a low-power, focused cauterization. Using this standardized low-power protocol under continuous irrigation, the aneurysm tissue was seen to shrink gradually and reliably during coagulation, facilitating subsequent clipping, and no instances of aneurysm wall rupture were encountered during bipolar use. The bipolar coagulation was applied intermittently (in the short bursts of a few seconds) rather than continuously, allowing time for thermal diffusion and tissue response while the temporary clip remained in place. After each bipolar application, the temporary clip was removed to re-perfuse the parent artery, and the aneurysm clipping was then completed or continued as per the standard microsurgical procedure.

### Learning curve and safety measures

The incorporation of bipolar coagulation into aneurysm surgery was done in a stepwise, safety-conscious manner. A single experienced cerebrovascular neurosurgeon started performing aneurysm surgery only after studying in a well-equipped neuroanatomy laboratory for 13 months (Baskaya Skull-base Lab, University of Wisconsin-Madison). We decided applying bipolar coagulation technique only after performing 30 ruptured aneurysm cases with standard microsurgical techniques described by Yasargil. These 30 cases established a baseline and allowed the surgical team to gain experience in the standard technique before introducing the new adjunct. In the next phase, bipolar cautery was introduced cautiously: in 7 subsequent cases, it was used only for post-clip dome remodeling (i.e., only after the aneurysm had been secured with a clip). Limiting the use to post-clip shrinkage first allowed the team to evaluate the safety of bipolar application on the aneurysm sac in a controlled setting, since the aneurysm was already occluded by the clip at that point.

After these initial trials demonstrated that bipolar coagulation could be applied without causing intraoperative complications, the technique was adopted more broadly. In the remaining cases of the series, bipolar coagulation was utilized in pre-clipping stages and/or in combination (both before and after clipping) whenever indicated by aneurysm morphology and intraoperative judgment. Over the course of the study, no intraoperative aneurysm ruptures were attributed to the use of bipolar cautery. This is consistent with previous reports suggesting that bipolar use, when performed with proper technique, does not substantially increase rupture risk. We proactively monitored for any signs of aneurysm wall thinning or impending rupture during coagulation, and none were observed. The surgical team became progressively more confident in the technique as the series advanced, constituting a learning curve that emphasized meticulous technique (temporary clipping, irrigation, low power, blunt tips) to maximize safety. All cases were accompanied by standard intraoperative precautions such as readiness for immediate clip application in case of any aneurysm tear, a safe and readily available proximal control and a second suction was kept available to manage any bleeding promptly. No adverse events were specifically linked to the use of bipolar coagulation in this study.

### Postoperative imaging and follow-up protocol

Postoperative evaluation followed a standardized institutional protocol designed to ensure early detection of complications and confirmation of aneurysm occlusion. All patients underwent a non-contrast cranial CT and thin sliced CT angiography immediately after the operation. An additional DSA was performed in all cases within the first 24 h after surgery to ensure complete occlusion of the aneurysm and intact normal vasculature. All the patients underwent a follow-up DSA 6 months after the operation as the routine of our center.. Clinical follow-up examinations were scheduled at 3, 6, 12, and 24 months, with additional imaging performed when clinically indicated. Mortality was defined as any death occurring within the first 30 days following surgery.

### Statistical analysis

Statistical analyses were performed using IBM SPSS Statistics (version 22.0). Normality of continuous variables was assessed using the Shapiro–Wilk test. Non-parametric comparisons of continuous variables were conducted using the Kruskal–Wallis test, while categorical variables were analyzed using the Chi-square test or Fisher’s exact test as appropriate. Numerical data are presented as mean ± standard deviation (SD), and categorical data as frequencies and percentages. Given that most continuous variables were not normally distributed, non-parametric tests (Kruskal–Wallis) were used for group comparisons. A two-tailed *p*-value < 0.05 was considered statistically significant.

## Results

### Demographic and preoperative characteristics

A total of 50 patients who underwent aneurysm surgery with the adjunctive use of bipolar coagulation were included in this study. The mean age was 56.94 ± 12.3 years (range, 32–81 years), and the cohort consisted of 30 females (60.0%) and 20 males (40.0%). The most common aneurysm location was the anterior communicating artery (AcomA) in 24 patients (48.0%), followed by the middle cerebral artery (MCA) in 20 patients (40.0%). Other locations included the posterior communicating artery (PcomA) (4.0%), internal carotid artery bifurcation (2.0%), posterior inferior cerebellar artery (PICA) (2.0%), paraclinoidal segment (2.0%), and pericallosal artery (2.0%). Multiple aneurysms were present in 7 patients (14.0%), while the remaining 43 patients (86.0%) had a single aneurysm. In terms of morphology, 42 aneurysms (84.0%) were classified as irregular, and 8 (16.0%) as regular. A history of smoking was noted in 28 patients (56.0%), with a mean exposure of 42.5 ± 27.91 pack-years (range, 10–120), while 22 patients (44.0%) were non-smokers. Hypertension was present in 20 patients (40.0%), and diabetes mellitus was observed in 6 patients (12.0%). Regarding clinical presentation, the mean Fisher grade was 2.06 ± 1.48 (range, 0–4), and the mean preoperative WFNS grade was 1.6 ± 1.29 (range, 1–5). Aneurysmal subarachnoid hemorrhage was detected in 37 patients (74.0%) at the time of admission. For the ruptured cases, the mean timing from ictus to surgery was 2.22 ± 2.04 days (range, 0–9 days), reflecting a predominantly early-intervention strategy (Table 1).

**Table 1:** Demographic and preoperative clinical characteristics of patients undergoing aneurysm surgery with bipolar coagulation

Variable	*n* (%)/Mean ± SD (min–max)
Age	56.94 ± 12.3 (32.0–81.0)
Gender	F: 30 (60.0%) M: 20 (40.0%)
Aneurysm	AcomA: 24 (48.0%) MCA: 20 (40.0%) PcomA: 2 (4.0%) ICA bifurcation: 1 (2.0%) PICA: 1 (2.0%) Paraclinoidal: 1 (2.0%) Pericallosal Aretery: 1 (2.0%)
Multiple	No: 43 (86.0%) Yes: 7 (14.0%)
Side	Right: 37 (74.0%) Left: 12 (24.0%) Median: 1 (2.0%)
Shape	Irregular: 42 (84.0%) Regular: 8 (16.0%)
Smoke (pack-year)	No: 22 (44.0%) Yes: 28 (56.0%) Yes: 42.5 ± 27.91 (10.0–120.0)
Hypertansion	No: 30 (60.0%) Yes: 20 (40.0%)
Diabetes Mellitus	No: 44 (88.0%) Yes: 6 (12.0%)
Fisher	2.06 ± 1.48 (0–4)
Preoperative WFNS	1.6 ± 1.29 (1–5)
Bleeding	Yes: 37 (74.0%) No: 13 (26.0%)
Operation Timing (day)	2.22 ± 2.04 (0–9)

*SD* Standard deviation; min–max: minimum–maximum values

### Intraoperative findings and surgical technique overview

Among the 50 patients, the pterional approach was the most frequently used surgical corridor (*n* = 47, 94.0%), while anterior interhemispheric, frontotemporal, and suboccipital approaches were each utilized in one patient (2.0% each). The mean number of aneurysm clips applied was 1.6 ± 0.95 (range: 0–5), and the mean aneurysm size was 6.43 ± 2.43 mm (range: 1.5–14.0 mm).

Bipolar coagulation was employed for neck remodeling in 31 patients (62.0%) and for dome coagulation in 30 patients (60.0%). Coagulation following clip application was required in 29 patients (58.0%), and post-puncture coagulation was performed in 9 cases (18.0%). Sole reliance on coagulation combined with cotton wrapping was limited to 1 patient which was a small (3 mm) posterior communicating artery aneurysm (2.0%).

Dome excision and dome puncture were conducted in 14 (28.0%) and 15 patients (30.0%), respectively. “Baby aneurysm” morphology was noted in 13 patients (26.0%). No instances of intraoperative rupture were encountered (0%). Intraoperative oozing occurred during the aneurysm dissection in 11 cases (22.0%) all of which were easily controlled. Lamina terminalis fenestration was performed in 28 patients (56.0%), and gyrus rectus resection was necessary in 15 cases (30.0%). Retractor, which was only used for the holding purpose, was used in 32 surgeries (64.0%).

Detailed analysis of the indications for bipolar coagulation revealed its use in neck remodeling prior to clip application in 15 patients (30.0%), identification of residual aneurysm post-clipping in 14 patients (28.0%), dome dissection from parent arteries in 13 patients (26.0%), and neck dissection from adjacent vessels in 5 patients (10.0%). Additional indications included pre-wrapping coagulation in 2 cases (4.0%) and repair of a partially damaged patent artery in 1 case (2.0%) (Table 2).

**Table 2:** Intraoperative surgical details and bipolar coagulation utilization during aneurysm clipping procedures

Variable	*n* (%)/Mean ± SD (min–max)
Approach	Pterional: 47 (94.0%) Anterior interhemispheric: 1 (2.0%) Frontotemporal: 1 (2.0%) Suboccipital: 1 (2.0%)
Clip count	1.6 ± 0.95 (0–5)
Size (mm)	6.43 ± 2.43 (1.5–14.0)
Neck Coagulation	Yes: 31 (62.0%) | No: 19 (38.0%)
Dome Coagulation	Yes: 30 (60.0%) | No: 20 (40.0%)
Post-Clip Coagulation	Yes: 29 (58.0%) | No: 21 (42.0%)
Post-puncture Coagulation	No: 41 (82.0%) | Yes: 9 (18.0%)
Only coagulation-Cotton Wrapping	No: 49 (98.0%) Yes: 1 (2.0%)
Dome Excision	No: 36 (72.0%) | Yes: 14 (28.0%)
Dome Puncture	No: 35 (70.0%) | Yes: 15 (30.0%)
Baby Aneurysm	No: 37 (74.0%) | Yes: 13 (26.0%)
Early Rupture	No: 50 (100.0%)
Intraoperative Oozing	No: 39 (78.0%) | Yes: 11 (22.0%)
Lamina Terminalis opening	Yes: 28 (56.0%) | No: 22 (44.0%)
Gyrus Rectus Resection	No: 35 (70.0%) | Yes: 15 (30.0%)
Retractor Use	Yes: 32 (64.0%) | No: 18 (36.0%)
Bipolar Coagulation Utiliity	Remodeling neck for clipping 15 (30.0%) Check the Residual aneurysm after clipping 14 (28.0%) Dome dissection from the artery 13 (26.0%) Neck dissection from the artery 5 (10.0%) Coagulation of the aneurysm before wrapping 2 (4.0%) Patent artery repair 1 (2.0%)

*WFNS* World Federation of Neurosurgical Societies scale, *SD* Standard deviation; min–max: minimum–maximum values

### Postoperative outcomes and follow-up results

At the time of discharge, the mean WFNS grade had improved to 1.38 ± 1.07 (range: 1–5). In postoperative DSA residual aneurysm was detected in only 2 patients (4.0%), indicating a complete obliteration rate of 96.0%. One of these patients was reoperated the next day and the aneurysm was completely obliterated by clip reconstruction. Other patient with residual aneurysm rejected surgery and underwent endovascular treatment in another center. Overall mortality during follow-up was 6.0% (*n* = 3), with the remaining 47 patients (94.0%) surviving beyond the acute postoperative period (Table 3). No death was related to surgical technique or any inadvertent injury to any neurovascular structure. All 3 deaths were due to vasospasm which couldn’t be resolved despite the application of all treatment modalities.

**Table 3:** Postoperative outcomes and follow-up data

Variable	*n* (%)/Mean ± SD (min–max)
Residue Anerysm	No: 48 (96.0%) | Yes: 2 (4.0%)
Discharge WFNS	1.38 ± 1.07 (1–5)
Follow-up (month)	13.99 ± 6.67 (2.4–32.3)
Exitus	No: 47 (94.0%) | Yes: 3 (6.0%)

Intraoperative bipolar coagulation techniques were categorized into four groups based on the primary anatomical target and purpose of application: neck-focused coagulation (*n* = 20) for neck remodeling or dissection adjacent to parent arteries, dome-focused coagulation (*n* = 29) for dome reduction, residual dome shrinkage, post-clip coagulation of already clipped sac to expose exact clip position or dissection near distal branches, and repair-oriented coagulation (*n* = 1) used for arterial wall injury repair.

Preoperative WFNS scores showed no significant difference among the groups (1.4 ± 1.1 in neck-focused, 1.69 ± 1.42 in dome-focused, and 3.0 in the repair group; *p* = 0.20). Similarly, discharge WFNS scores were comparable (1.25 ± 0.91, 1.48 ± 1.18, and 1.0, respectively; *p* = 0.71). The number of clips used did not differ significantly (mean 1.5 in neck-focused vs. 1.69 in dome-focused vs. 1.0 in repair group; *p* = 0.56).

Residual aneurysm was noted in one case (5.0%) in the neck-focused group and the other in the post-clip group, but none in the dome-focused group. Early rupture was not encountered in any patient. Mortality was low and statistically nonsignificant across groups (1/20 [5.0%] in neck-focused, 2/29 [6.9%] in dome-focused, and 0/1 in repair; *p* = 0.93).

The indications for bipolar coagulation varied: neck-focused usage primarily targeted neck remodeling for clipping (75.0%) and neck dissection (25.0%); dome-focused cases mostly included remnant dome management (48.3%), dome dissection (44.8%), and coagulation before wrapping (6.9%). The single repair case involved arterial wall repair due to intraoperative injury. Despite this variability in indications, statistical comparison among the groups did not show a significant difference (*p* = 5.45) (Table 4).

**Table 4:** Comparison of intraoperative bipolar coagulation strategies based on targeted aneurysm regions: neck-focused, dome-focused, and repair-oriented techniques

Variable	Neck-focused	Dome-focused	Other/repair	*p*_value
Preoperative WFNS	1.4 ± 1.1 (1–5)	1.69 ± 1.42 (1–5)	3.0 ± nan (3–3)	0.20*
Discharge WFNS	1.25 ± 0.91 (1–5)	1.48 ± 1.18 (1–5)	1.0 ± nan (1–1)	0.71*
Clip count	1.5 ± 0.83 (1–4)	1.69 ± 1.04 (0–5)	1.0 ± nan (1–1)	0.56*
Intraoperative Oozing	4 (20.0%)	6 (20.7%)	1 (100.0%)	0.16**
Exitus	1 (5.0%)	2 (6.9%)	0 (0.0%)	0.93**
Residual Anerysm	1 (5.0%)	0 (0.0%)	1 (100.0%)	3.27**
Early Rupture	0 (0.0%)	0 (0.0%)	0 (0.0%)	1.0**
Reason for bipolar coagulation	Remodeling neck for clipping: 15 (75.0%) Neck dissection from the artery: 5 (25.0%)	Check the Residual aneurysm after clipping: 14 (48.3%) Dome dissection from the artery: 13 (44.8%) Coagulation of the aneurysm before wrapping: 2 (6.9%)	Patent artery repair: 1 (100.0%)	5.45**

*WFNS* World Federation of Neurosurgical Societies scale, *SD* Standard deviation; min–max: minimum–maximum values. *p*-values calculated using Kruskal–Wallis test for continuous variables (*) and Chi-square test for categorical variables (**)

## Discussion

In this single-surgeon series of 50 aneurysm clippings with adjunctive bipolar coagulation, we observed no bipolar-related intraoperative ruptures, a 96% early complete obliteration rate, and no ischemic complications attributable to bipolar use. Bipolar was most commonly employed for neck remodeling and dome reduction in morphologically challenging aneurysms. Bipolar coagulation has played an important role in cerebrovascular microneurosurgery, evolving from early electrothermic concepts into a useful adjunct in selected complex aneurysm cases [11]. Greenwood’s introduction of “two-point” coagulation in 1940 and Malis’ subsequent development of a dedicated bipolar generator, which reduced thermal spread and tissue injury, marked key early milestones [5, 13]. These innovations enabled the pioneering microsurgical work of Yasargil in the 1960s, who integrated both microscope and bipolar coagulation into precise vascular reconstruction and aneurysm surgery [19]. His emphasis on focused, brief bursts—guided by suction and delivered under high magnification and illumination—set the foundation for the modern microsurgical ethos of “simple, clean, fast, and anatomically preserving.” Over time, refinements such as “short-burst” method further increased precision and safety, making bipolar coagulation a predictable and controlled maneuver in cerebrovascular surgery [9, 14, 17, 20].

Despite advances in clip technology, microsurgical clipping still poses significant challenges when aneurysm morphology obscures the neck or restricts surgical freedom [1, 6]. Broad-based necks, multilobulated morphology, and bulbous domes may hinder visualization [10]. Bulging domes in particular compromise exposure of branching or perforating arteries, thus increasing the risk of suboptimal clip placement [21]. Moreover, fragile walls or thin blebs introduce rupture risk during dissection [9], while calcified or atherosclerotic necks can resist clip closure [19]. These anatomical and pathological limitations have driven the integration of adjunct techniques—including temporary vessel occlusion, clip reconstruction strategies, and bipolar remodeling—to improve access and achieve durable occlusion [2].

Among these adjunctive maneuvers, dome coagulation remodeling has emerged as a useful technique for reducing aneurysm volume and clarifying the neck [17, 19]. Controlled bipolar pulses applied to the mid-dome can contract and thicken the wall, thereby converting an obstructive dome into a more manageable profile. This facilitates clip placement in small- to medium-sized saccular aneurysms with limited visibility. Similarly, post-clip bipolar coagulation can obliterate residual dome or neck remnants and separate the sac from adjacent perforators [2, 4, 15, 16].

Historical techniques also contribute to modern practice. Sano’s “clip-and-shrink” method demonstrated that dome puncture followed by bipolar coagulation can collapse the sac after a secure clip is applied; when clipping is incomplete, controlled shrinkage may allow safe repositioning. However, Sano cautioned that coagulated tissue undergoes degenerative changes, emphasizing the need for judicious use [17]. Likewise, Hernesniemi frequently “killed the aneurysm” by cauterizing and excising the clipped dome to eliminate mass effect and ensure complete occlusion [6]. Another valuable adjunct is the cotton-assisted technique, where cotton or Surgicel reinforces a fragile dome or neck tear and distributes heat during bipolar application, often securing remnants that are not amenable to clip reconstruction [16].

Safe bipolar use hinges on proper power settings and strict microsurgical control. The modern short-burst technique— < 1-s bursts, immediate release, continuous irrigation—minimizes thermal accumulation and prevents sticking [4, 12, 18]. Yasargil recommended moist bipolar tips, frequent cleaning, and moderate power (~ 25 Malis units) for controlled dome shrinkage [21]. Recent studies report operating at only 10–20% of maximum power (~ 3–5 W) for dome reduction [8]. Using fine 0.2–0.3 mm tips for delicate regions and slightly blunter tips for thicker wall segments, short bursts at 20–25 Malis units (≈3–5 W) can remodel the sac without compromising arterial flow [4]. Continuous saline irrigation, non-stick bayonet forceps, and temporary parent-artery clipping further enhance control and safety [8, 21]. In our bipolar setting (Aesculap GN060; Aesculap, Tuttlingen, Germany) we use 7 unit and find it very effective.

Outcome data support the selective use of bipolar coagulation. In middle cerebral artery aneurysms, dome remodeling markedly improved neck exposure and enabled safe clipping without intraoperative ruptures or added complications [10]. The Helsinki group’s long-term experience demonstrated that short-burst bipolar repair reliably sealed small wall tears while preserving vessel patency—validated by micro-Doppler and intraoperative fluorescence angiography [3, 4]. However, excessive or prolonged coagulation may precipitate rupture, and most authors caution against routine or indiscriminate use, especially in heavily calcified or giant aneurysms [12].

Another important point to emphasize is that bipolar coagulation of the aneurysm dome and/or neck can reduce the number of clips required for the sac closure. When fewer clips are needed, they are less likely to hamper the exposure and less likely to obscure the surrounding anatomy, clip blades and perforating vessels. Avoiding clip crowding and maintaining an unobstructed field consequently contribute to a safer and more reliable clip application.

Our study adds to this literature by providing a detailed description of how bipolar coagulation is used in routine microsurgical practice. By incorporating dome and neck coagulation patterns, post-puncture coagulation, dome excision, cotton-wrapping/coating, rupture timing, clip number, and other operative variables, our dataset offers a practical overview of real operative use. Importantly, our findings demonstrate that when employed selectively and with precise micro-technique, bipolar coagulation contributed to safer clipping, improved surgical exposure, and a high rate of complete obliteration, with no observed increase in procedure-related morbidity. The distribution of coagulation patterns in our series underscores that the technique is applied for diverse but well-defined intraoperative purposes.

Our findings suggest that bipolar coagulation may represent a safe adjunct in selected aneurysm morphologies when applied with strict microsurgical technique. In our cohort, bipolar use—whether pre-clip, post-clip, or both—was associated with improved visualization of the neck, as documented in operative reports and facilitated clip application in anatomically challenging aneurysms, without causing any intraoperative ruptures attributable to cautery. Notably, complete aneurysm obliteration on early postoperative imaging was achieved at a high rate, and no patient demonstrated clip migration, parent artery stenosis, or ischemic complications related to bipolar use. The absence of bipolar-related rupture in our series is notable, as fear of unintended perforation remains one of the primary reasons many surgeons avoid this technique. Moreover, bipolar remodeling was associated with improved visualization in selected anatomically challenging cases in this cohort. Our results suggest that, when performed with low power, intermittent bursts, and strict micro-technique, bipolar coagulation can serve as an additional option in selected difficult anatomical configurations by improving surgical exposure and contributing to durable aneurysm closure without increasing procedural risk.

Our findings, when considered in the context of historical and contemporary literature, support the continued role of bipolar coagulation as a selective adjunct in aneurysm surgery [11]. Its optimal use requires careful patient selection, anatomical insight, and disciplined microsurgical execution. When applied selectively and with meticulous technique, bipolar coagulation may serve as a useful adjunct during aneurysm clipping in selected cases.

### Limitations

This study has several important limitations that should be acknowledged. First, its retrospective design inherently limits causal inference and is subject to selection bias. Only cases in which bipolar coagulation was deliberately employed were included, which likely represents a subset of aneurysms with more complex or unfavorable morphology. Second, this is a single-center, single-surgeon experience. Although this approach minimizes inter-operator variability and allows for a consistent surgical philosophy and technique, it also limits external validity. Third, the absence of a control group of aneurysms clipped without bipolar coagulation precludes direct comparison and prevents definitive conclusions regarding the superiority of bipolar-assisted techniques over standard clipping alone. Fourth, although a stepwise learning curve was described, the study design does not allow for formal analysis of learning effects or temporal outcome differences. Improvements in technique and confidence over time may have influenced intraoperative decision-making and outcomes. Finally, long-term angiographic follow-up beyond 24 months was not available for all patients. Therefore, late aneurysm recurrence or delayed vessel-related complications could not be fully assessed. Despite these limitations, the study provides a detailed and systematic description of real-world bipolar coagulation use in microsurgical aneurysm clipping and may serve as a foundation for future prospective and comparative investigations.

## Supplementary Information

Below is the link to the electronic supplementary material.

ESM 1 Supplementary Material 1 (MP4 315 MB)

ESM 2 Supplementary Material 2 (MP4 409 MB)

ESM 3 Supplementary Material 3 (MP4 330 MB)

ESM 4 Supplementary Material 4 (MP4 376 MB)

ESM 5 Supplementary Material 5 (MP4 268 MB)

ESM 6 Supplementary Material 6 (MP4 87.5 MB)

## Data Availability

The datasets generated and/or analyzed during the current study are available from the corresponding author upon reasonable request.

## Abbreviations

AcomA: Anterior Communicating Artery
CTA: Computed Tomography Angiography
CT: Computed Tomography
ICA: Internal Carotid Artery
MCA: Middle Cerebral Artery
PcomA: Posterior Communicating Artery
PICA: Posterior Inferior Cerebellar Artery
SD: Standard Deviation
WFNS: World Federation of Neurosurgical Societies
